# Plant endophytic fungi against plant-parasitic nematodes: from direct antagonism to plant-mediated biocontrol

**DOI:** 10.3389/fpls.2026.2002520

**Published:** 2026-10-09

**Authors:** María Lorena Giachero, Nathalie Marquez, Stephane Declerck

**Affiliations:** 1 Earth and Life Institute - Applied Microbiology (ELIM), Université Catholique de Louvain, Louvain-la-Neuve, Belgium; 2 Instituto de Patología Vegetal (IPAVE), Centro de Investigaciones Agropecuarias, Instituto Nacional de Tecnología Agropecuaria, Córdoba, Argentina; 3 Unidad de Fitopatología y Modelización Agrícola (UFyMA), CONICET, Córdoba, Argentina

**Keywords:** biological control, crop protection, endophyte, induced systemic resistance, nematodes, plant immunity, sustainable agriculture

## Abstract

Plants have evolved sophisticated and tightly regulated immune systems to defend themselves against pathogen, whereas pathogens have developed diverse strategies to evade or manipulate host defenses. Plant-parasitic nematodes pose a particular challenge because of their intimate and prolonged interactions with host tissues, during which they actively manipulate plant physiology and immune responses. The limitations of conventional nematode control strategies have increased interest in plant-associated microorganisms as sustainable alternatives. Among these, endophytic fungi are emerging as promising allies because they can act through complementary mechanisms, combining direct antagonism of nematodes with the ability to modulate and prime plant immunity. This review integrates current knowledge on plant-nematode interactions with emerging insights into the role of endophytic fungi as modulators of plant defense. We examine how fungal endophytes influence hormone signaling, defense-related pathways, systemic resistance, and other components of plant immunity, while also considering their direct effects on nematode survival, development, and reproduction. We demonstrate that the protective potential of endophytic fungi extends beyond direct biocontrol and involves a broader reprogramming of plant responses that can enhance resistance to nematode attack. Finally, we discuss the factors that determine the consistency of these interactions, including fungal and plant genotype, environmental conditions, and the physiological and ecological context of the plant–fungus–nematode interaction. Understanding these mechanisms will be critical for translating endophyte-mediated plant protection into reliable and sustainable strategies for nematode management.

## Introduction

### Plant-parasitic nematodes: importance and host interactions

Plant-parasitic nematodes (PPN) are major constraint to global agricultural productivity, causing substantial yield losses in a wide range of cropping systems (Conceição, 2025). Economically and scientifically important groups include the Genera *Meloidogyne, Heterodera, Globodera, Pratylenchus, Radopholus, Ditylenchus, Bursaphelenchus, Rotylenchulus, Xiphinema, Nacobbus*, and *Aphelenchoides* (Jones et al., 2013). Among these, root–knot nematodes (RKN; e.g., *Meloidogyne* spp.), cyst nematodes (CN; e.g., *Heterodera* spp., *Globodera* spp.) and root-lesion nematodes (RLN; e.g., *Pratylenchus* spp.), are among the most damaging groups because of their broad host ranges, persistence in agricultural soils, and capacity to cause severe root dysfunction.

Populations of PPN differ markedly in their geographic distribution, host range, and parasitic specialization. Some species, including several *Meloidogyne* spp., have a near-cosmopolitan distribution, whereas others have more restricted geographic range or highly specific host association. For example, *Heterodera carotae*, is primarily associated with carrot production. The diversity and composition of PPN communities also vary considerably among crops. While some crops are affected by a relatively limited number of species, others, for instance sugarcane and rice, can host diverse assemblages of nematode genera and species, increasing the complexity of disease diagnosis and management (Nicol et al., 2011).

The diversity of PPN and their complex interactions with their hosts make sustainable management particularly challenging. Integrated Nematode Management (INM) therefore relies on the combination of complementary approaches, including cultural practices, resistant cultivars, biological control, targeted and restricted use of nematicides (Sikora et al., 2023; Maleita et al., 2023). Within this framework, increasing attention is being given to beneficial microorganisms capable of interfering with nematode infection while simultaneously enhancing plant defense.

In this review, we aim to integrate current knowledge on plant-nematode interactions with emerging insights into endophytic fungi as modulators of plant immunity, highlighting their dual role in direct antagonism and indirect defense priming. Understanding the molecular and ecological basis of these interactions is therefore essential for exploiting endophytic fungi as reliable components of sustainable PPN management.

### Plant-nematode interaction and feeding strategies

The interaction between PPN and their host plants begins in the soil, where chemical cues released by roots contribute to host location and recognition. Root exudates play a crucial role in host-nematode interactions. They can stimulate the hatching of cysts and eggs and influence the attraction and orientation of juveniles, particularly second-stage juveniles (J2) of RKN and CN (Bell et al., 2019). These chemical signals therefore represent an important interface between plants and nematodes and contribute to determining the outcome of the interaction.

Successful parasitism requires nematodes to perceive and manipulate host signaling processes. For example, Weerasinghe et al. (2005) demonstrated that RKN can induce early signaling events in plant roots that partially overlap with pathways involved in beneficial plant–microbe interactions including responses associated with Nod factors signaling produced by nitrogen-fixing bacteria. This observation suggest that nematodes may exploit pre-existing plant signaling networks to establish compatible interactions (Weerasinghe et al., 2005). However, current evidence indicates that the relationship is more complex than a simple hijacking of symbiotic signaling. Nematode infection involves the convergence of conserved plant signaling pathways with parasite-specific mechanisms, including the secretion of effectors that reprogram host plant responses (Costa et al., 2020).

A defining adaptation of PPN is the stylet, a specialized protrusible feeding structure that enables nematodes to pierce cell walls and access cellular contents. Stylet activity is coordinated with secretions produced by specialized esophageal gland cells, which deliver proteins and other molecules into host tissues and contribute to feeding, migration, and manipulation of host cells (Hussey, 1989; Davis et al., 2004; Jones et al., 2013). The extent and nature of root invasion differ substantially among PPN and form the basis for their classification into distinct feeding strategies.

Ectoparasitic nematodes, such as *Belonolaimus, Longidorus* and *Xiphinema* remain outside the root and use their stylet to feed on cells of the epidermis and outer cortical tissues. In contrast, migratory endoparasites, including *Pratylenchus, Radopholus, Ditylenchus, Bursaphelenchus, Aphelenchoides, Hirschmanniella*, penetrate root tissues and migrate through and/or between cells, while feeding on successive cells. Their movement and feeding cause localized cell death, tissue disruption, and impairment of root functions. Semi-endoparasitic nematodes, including *Rotylenchulus, Nacobbus, Tylenchulus*, partially invades the root; the anterior region establishes a permanent feeding site while the posterior portion remains outside the root. By contrast, sedentary endoparasites, particularly CN such as *Heterodera, Globodera* and RKN such as *Meloidogyne* fully penetrate the root and induce the formation of a highly specialized and permanent feeding structure. These nematodes induce extensive developmental and transcriptional reprogramming of host cells. RKN stimulates the differentiation of multinucleate giant cells, whereas CN induces the formation of syncytia through the fusion and modification of neighboring cells. These highly specialized feeding structures function as sustained nutrient sinks that support nematode growth and reproduction (Bernard et al., 2017).

The contrasting feeding strategies of PPN are therefore associated with distinct patterns of tissue damage and host manipulation. Despite these differences, successful parasitism generally depends on the nematode’s capacity to overcome or redirect plant immune responses. This intimate connection between feeding-site establishment, immune suppression, and host physiological reprogramming provides a key framework for understanding how beneficial microorganisms—including endophytic fungi—may interfere with nematode infection and enhance plant resistance.

## Nematode effectors and the hijacking of plant immunity

### Effector proteins and virulence mechanisms

Plant roots perceive nematode-derived molecules and activate immune responses that can restrict infection. This was demonstrated by the recognition of conserved nematode-associated molecules, such as ascarosides, that can induce pattern-triggered immunity (PTI) and effector-triggered immunity (ETI) in plants (Manosalva et al., 2015; Holbein et al., 2016). However, successful parasitism requires PPNs to overcome, suppress, or redirect these defenses. To achieve this, nematodes secrete effector proteins through their stylet into host tissues, where they interfere with immune signaling, and reprogram host development and metabolism to create an environment conducive to feeding and reproduction (Bali and Gleason, 2024). The composition and functional complexity of these effector repertoires vary among PPN lineages and are closely associated with their feeding strategies and degree of host dependence (**Figure 1**).

**Figure 1:**
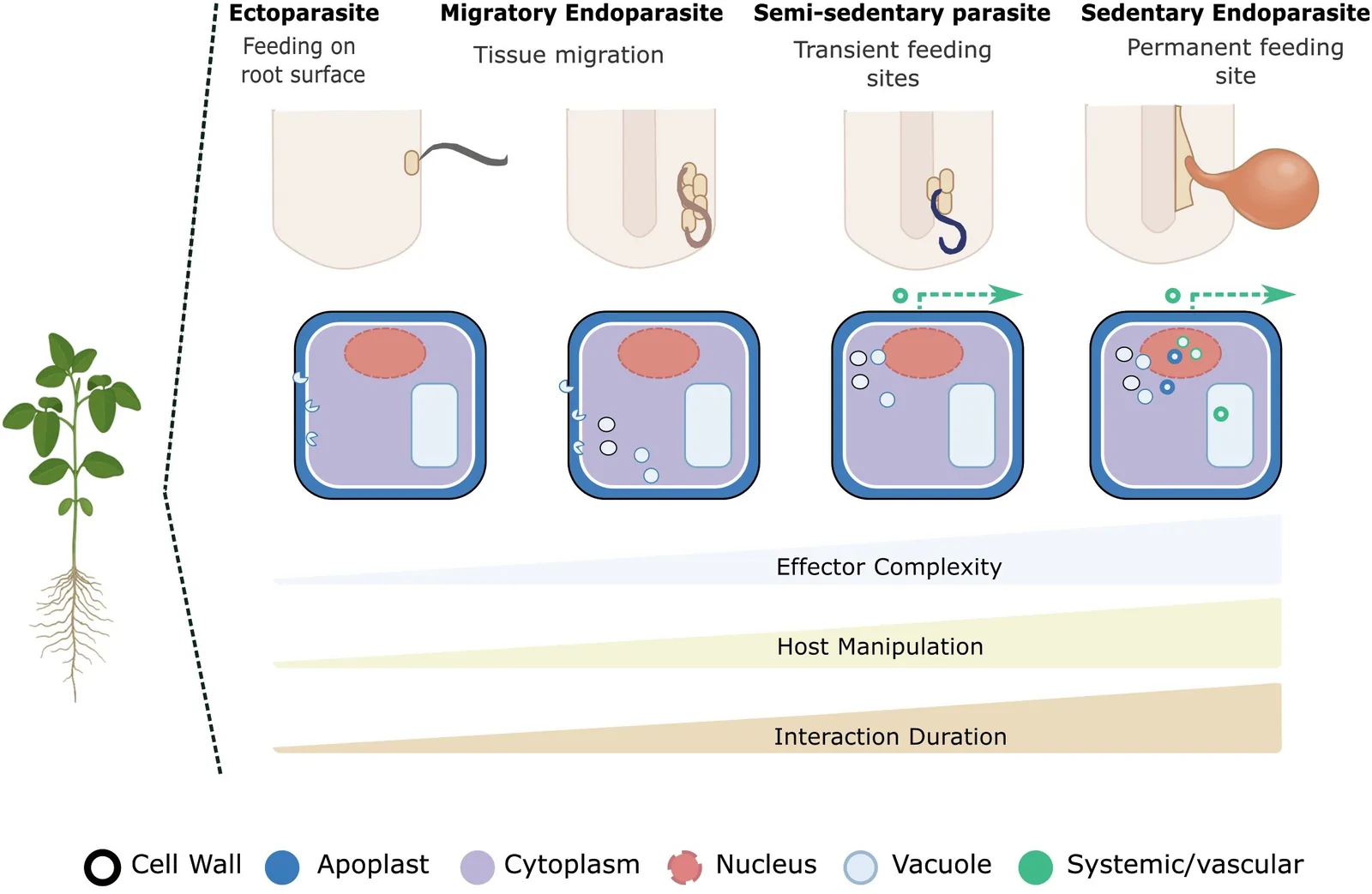
Schematic representation of the main strategies of plant invasion adopted by plant-parasitic nematodes (PPNs) — ectoparasites, migratory endoparasites, Semi-sedentary and sedentary endoparasites — and their associated levels of effector complexity, host manipulation, and interaction duration, illustrated using soybean (*Glycine max*) as the host plant model. Ectoparasites, such as *Xiphinema* and *Trichodorus* spp., feed exclusively on root surface cells without penetrating host tissues and generally exhibit relatively simple effector repertoires and brief host interactions. Migratory endoparasites, including *Pratylenchus* and *Radopholus* spp., invade root cortical tissues across multiple life stages but do not establish permanent feeding sites, relying on a moderately complex set of effectors involved in cell wall degradation and suppression of basal host defense. Semi-sedentary nematodes, such as *Rotylenchulus reniformis and Nacobbus aberrans*, establish transient feeding sites and display moderately expanded effector repertoires. Sedentary endoparasites, represented by root-knot nematodes (*Meloidogyne* spp.) and cyst nematodes (*Heterodera* and *Globodera* spp.), deploy highly sophisticated effector arsenals, targeting multiple layers of plant immunity — including PTI, ETI, and hormone signaling pathways — to establish and maintain permanent feeding sites. The gradients indicate the relative increase in effector complexity, extent of host manipulation, and duration of plant-nematode interactions from ectoparasites to sedentary endoparasites. Created in https://BioRender.com.

Sedentary endoparasites have evolved particularly sophisticated and temporally regulated effector repertoires that not only facilitate host invasion but also reprogram plant cell fate, leading to the formation and maintenance of specialized feeding structures such as giant cells and syncytia (Mitchum et al., 2013; Ali et al., 2017). These processes involve extensive modulation of host transcriptional networks, hormone signaling pathways, and immune responses (Hewezi and Baum, 2013; Yusuf and Bello, 2025), and are accompanied by profound morphological and physiological changes within the feeding sites. Thus, nematode effectors function not merely as virulence factors but as molecular tools for redirecting plant developmental programs toward the formation of highly specialized nutrient sinks.

A particularly remarkable example of this strategy is the molecular mimicry of endogenous plant signaling peptides. CLE-like effectors produced by nematodes belonging to the genera *Heterodera* and *Globodera*, mimic members of the plant CLAVATA3/ESR-related peptide family and contribute to the initiation, and maintenance of nematodes feeding sites by manipulating cell proliferation and differentiation (Mitchum et al., 2012; Replogle et al., 2013). Other peptide-like effectors target distinct host signaling pathways. CEP-like effector can interfere with peptide-mediated nitrogen signaling, whereas RALF-like effectors activate the FERONIA receptor kinase at the plasma membrane, and promote changes in the apoplast that attenuate pattern-triggered immune responses (Zhang et al., 2020; Yusuf et al., 2026). Such molecular mimicry illustrates the remarkable ability of sedentary nematodes to exploit conserved plant signaling systems that normally regulate development, nutrient allocation, and immunity. Beyond peptide mimicry, nematode effectors can also target central metabolic and protein-regulatory processes. Secreted chorismate mutases can alter host chorismate metabolism (the precursor for all aromatic amino acids) and downstream salicylic acid-associated defense pathways (Doyle and Lambert, 2003). Other effectors interfere with the ubiquitin-proteasome system, thereby altering the stability and activity of host proteins involved in defense signaling (Chronis et al., 2013).

In contrast, migratory endoparasites including *Pratylenchus* and *Radopholus* spp., rely predominantly on cell wall-degrading enzymes and a more limited set of immune modulators to support tissue penetration and intercellular movement, without establishing permanent feeding sites (Lu et al., 2024).

Semi-sedentary nematodes, such as *Rotylenchulus reniformis and Nacobbus aberrans*, occupy an intermediate position. They establish feeding sites that require host-cell modification but do not necessarily involve the extensive and permanent cellular reprogramming observed in sedentary endoparasites. Their effector repertoire includes cell wall-modifying enzymes, defense suppressors, and peptide-like effectors. For example, CEP-like effectors identified in *R. reniformis* have been proposed to manipulate systemic nitrogen signaling through the host vascular system (Eves-van den Akker et al., 2016).

Ectoparasitic nematodes have traditionally been considered to relying predominantly on mechanical feeding, with comparatively limited biochemical manipulation of host tissues. This view, however, may partly reflect pronounced research toward sedentary endoparasites, whose complex feeding sites and economic importance have driven extensive molecular and functional characterization of their secreted proteins (Mitchum et al., 2013), while comparable data for ectoparasitic clades remains comparatively limited. Nevertheless, transcriptomic analysis of species such as *Xiphinema index* and *Longidorus elongatus*, have identified dozens of transcripts encoding cell wall-degrading enzymes, including proteins potentially acquired through horizontal gen transfer (Danchin et al., 2017). These findings indicate that ectoparasitic nematodes also engage in active biochemical modification of the host and that their feeding strategy cannot be reduced to mechanical disruption alone.

The diversity of effector repertoire across PPN highlights a fundamental trade-off between the depth of host manipulation and the duration of the parasitic interaction. The need for extensive and sustained manipulation is greatest in sedentary endoparasites, which must establish and maintain a permanent nutrient-rich feeding structure, whereas migratory and ectoparasitic species interact with individual host cells for shorter periods. Effector evolution may consequently be shaped by ecological and physiological requirements associated with the duration, location, and nutritional demands of parasitism (Mitchum et al., 2013; Molloy et al., 2024).

Cell wall-modifying enzymes provide a common mechanistic link across these parasitic strategies. Cellulases, pectinases and related enzymes facilitate tissue invasion and migration but can also contribute to the structural remodelling of host roots. In sedentary nematodes, this activity is coupled with extensive metabolic reprogramming of host cells, resulting in the formation of a highly-specialized feeding site that sustains long-term parasitism. In semi-sedentary species, host cell reprogramming is generally less extensive and is maintained only for the duration of the juvenile stages before the nematode resume mobility (Eves-van den Akker et al., 2014; Wubben et al., 2015). Together, these coordinated mechanical and molecular processes transform the root into a highly permissive environment in which nematodes can evade host defences, acquire nutrients and complete their life cycle (Ali et al., 2017).

### Activation and manipulation of host defense responses

The outcome of plant–nematode interactions reflects a continuous molecular conflict between host immune activation and parasite-mediated immune suppression. Upon nematode attack, plants activate multiple layers of defense. In turn, PPNs deploy effectors that target key components of these pathways to attenuate immunity and promote parasitism.

Recent studies have revealed that these effectors can interfere simultaneously with multiple defense processes (**Figure 2B**). For instance, the RKN effector MiV86 promotes parasitism by manipulating the host ubiquitin–proteasome system. MiV86 stabilizes the host E3 ubiquitin ligase NbRNF217, thereby enhancing ubiquitination and subsequent degradation of the immune receptor NRC4, a key component of NLR-mediated defense responses. By targeting this intracellular immune hub, MiV86 suppresses ETI, and facilitate nematode infection and establishment of feeding site (Wu and Han, 2025; Qin et al., 2025). This example illustrates how a single nematode effector can simultaneously dampen basal defenses and target key intracellular signaling hubs to facilitate parasitic establishment.

**Figure 2:**
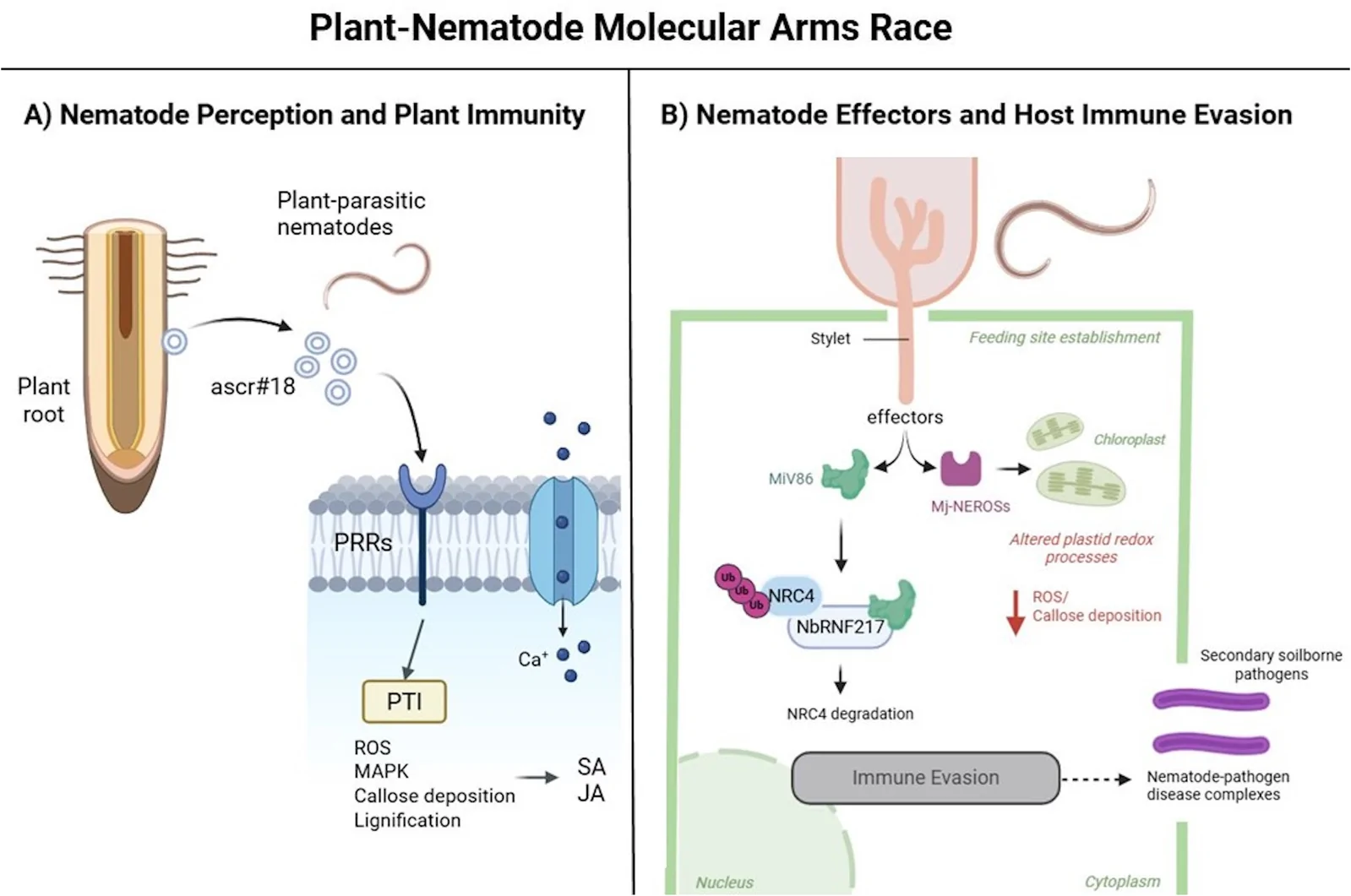
Molecular arms race between plants and plant-parasitic nematodes. **(A)** Nematode perception at the root. Conserved nematode-derived signaling molecules, such as the ascaroside ascr#18, are released by plant-parasitic nematodes and recognized by pattern recognition receptors (PRRs) at the plant plasma membrane. This perception triggers a Ca^2+^ influx and activates pattern-triggered immunity (PTI), including ROS production, MAPK signaling, callose deposition, and lignification. These defense responses converge on the activation of salicylic acid (SA) and jasmonic acid (JA) signaling pathways. **(B)** Suppression of host immunity by root-knot nematode (RKN) effectors during feeding-site establishment. The effector MiV86 stabilizes the E3 ubiquitin ligase NbRNF217, thereby promoting the ubiquitination and degradation of the intracellular immune receptor NRC4 and suppressing effector-triggered immunity (ETI). In parallel, the effector Mj-NEROSs targets chloroplasts, alters the redox state of the cytochrome b6f complex, and reduces ROS production and callose deposition. Together, these effectors promote broad immune evasion and facilitate nematode colonization. By compromising host defense, they may also increase susceptibility to secondary soilborne pathogens, potentially contributing to the development of nematode-pathogen disease complexes. Created in https://BioRender.com.

Nematode effectors can also target plastid-associated processes that contribute to immune signaling. The *Meloidogyne javanica* effector Mj-NEROSs interacts with the Rieske iron-sulfur protein (ISP), a component of the plastid cytochrome b6f complex, thereby altering plastid-associated redox processes (Stojilković et al., 2024). Functional analysis revealed that Mj-NEROSs expression in plant cells suppresses INF1-induced cell death, flg22-triggered callose deposition, and apoplastic ROS production, while transcriptomic analyses reveal the downregulation of numerous ROS-associated defense genes. These observations indicate that Mj-NEROSs can interfere with multiple immune outputs simultaneously.

Collectively, these findings reveal that RKN effectors can hijack plastid redox signaling as a strategy to suppress both PTI and cell death-associated layers to the immunosuppressive arsenal deployed during parasitic establishment (Stojilković et al., 2024).

The consequences of nematode-mediated immune modulation extend beyond the immediate interaction between the nematode and its host. Nematode feeding and migration can damage root tissues, generating entry points that can be readily exploited by other soilborne microorganisms. In addition, the formation of specialized feeding sites and associated metabolic reprogramming of host cells can generate a nutrient-rich microenvironment that favour microbial proliferation. Localized attenuation or reprogramming of host defenses may further increase susceptibility to secondary pathogens. These interactions can contribute to the emergence of nematode-pathogen disease complexes, in which co-infection produces more severe symptoms and yield losses than either organism alone (Blundell et al., 2026).

The dynamic interplay between plant defense activation and nematode immune suppression represents a central paradigm in the study of plant-nematode interaction (Goverse and Smant, 2014). Rather than being a unidirectional process, host immunity and nematode parasitism constitute a continuous and coevolutionary molecular dialogue in which the activation of plant defense and its suppression by nematode effectors are two inseparable and reciprocally driven processes. This framework, explicitly transposed from the zigzag model of Jones and Dangl (2006) to the nematode context by Goverse and Smant (2014), positions nematode effectors not merely as tools for invasion but as active and precise modulators of host immunity. Understanding plant defense responses in the presence of nematodes, therefore, requires simultaneous consideration of how plants sense and respond to nematode-derived signals, as well as how nematodes in turn deploy specialized effectors to counteract those responses and promote the cellular reprogramming necessary for successful parasitism. Understanding this balance is particularly relevant for identifying biological interventions capable of strengthening plant immunity while limiting the capacity of nematodes to suppress it.

### Nematode perception and activation of plant innate immunity

The Plant immune system operates through a series of sequential and interconnected defense layers that are deployed upon detection of potentially harmful organisms, including PPNs (**Figure 2A**). The first layer of inducible defense relies on cell surface-localized receptors that continuously sense the extracellular environment for molecular indicators of pathogen presence or host tissue damage. These receptors, collectively known as pattern recognition receptors (PRRs), detect two main categories of signals: conserved pathogen-derived molecules termed pathogen- or microbe-associated molecular patterns (MAMPs or PAMPs), and endogenous fragments released from damaged plant cells known as endogenous damage-associated molecular patterns (DAMPs). Recognition of these signals activate pattern-triggered immunity (PTI) which involves rapid calcium influx, production of reactive oxygen species (ROS), activation of mitogen-activated protein kinase (MAPK) cascades, cell-wall reinforcement through callose deposition and lignification, and extensive transcriptional reprogramming accompanied by changes in defense hormone signaling, including salicylic acid (SA) and jasmonic acid (JA) pathways (Jones and Dangl, 2006; Holbein et al., 2016; DeFalco and Zipfel, 2021; Ngou et al., 2022)). Should a pathogen successfully interfere with PTI through the action of secreted effectors, a second and more amplified immune layer is engaged. This response, designated ETI, depends on the intracellular recognition of effector proteins by nucleotide-binding leucine-rich repeat (NLR). The activation of these effectors can generate strong immune responses, including transcriptional reprogramming and, in some cases, localized programmed cell death or a hypersensitive response (HR), which functions to restrict pathogen spread (Holbein et al., 2016; DeFalco and Zipfel, 2021; Ngou et al., 2022).

Although the PTI-ETI framework is broadly applicable across plant interactions with bacteria, fungi, oomycetes, and viruses, the recognition of PPNs involves distinctive features that set this pathosystem apart. For microbial pathogens, the molecular identity of key PAMPs such as bacterial flagellin and lipopolysaccharides, or fungal chitin and glucans, and their corresponding PRRs have been well established over decades of research (Jones and Dangl, 2006; Holbein et al., 2016). By contrast, the molecular signatures through which plants detect the presence of nematodes remain far less understood, and only a handful of nematode-associated molecular patterns (NAMPs) have been identified and functionally validated. One of the best characterized examples is ascaroside #18 (ascr#18), a small nematode-derived signaling molecule conserved across multiple PPN species. Exogenous application of ascr#18 can prime immune responses in diverse plant species, activating MAPK signaling and promoting SA and JA-associated defense responses in both monocotyledonous and dicotyledonous plants (Manosalva et al., 2015). This finding demonstrates that plants can perceive chemical signatures associated with nematodes before or independently of direct recognition of nematode effectors.

The spatial and temporal dynamics of nematode perception may also differ from those of many microbial pathogens. Nematode-derived molecules can diffuse through the rhizosphere and reach root tissues before physical penetration, potentially allowing plants to initiate defense responses before direct contact with the invading organism (Chen et al., 2024). Finally, a notable gap exists in our understanding of ETI in this pathosystem: while NLR-mediated resistance has been functionally linked to the recognition of CN effectors in several crop species, the molecular events underpinning ETI against RKN remain largely unresolved, highlighting a key area for future investigation (Zheng et al., 2021; Chen et al., 2024).

## Control strategies and limitations

Plant parasitic nematodes are estimated to cause annual yield losses of approximately 12%-20% in major crops, corresponding to global economic losses of USD 157–175 billion (Khan, 2023; Ayaz et al., 2024). These estimates may nevertheless underestimate the true impact of PPNs because their damage is predominantly subterranean and can remain unnoticed until substantial root injury, yield reduction, or secondary disease has occurred. The most economically damaging genera include *Meloidogyne, Pratylnchus, Heterodera*, and *Globodera*, which collectively affect a broad range of food and cash crops across diverse agroecosystems (Jones et al., 2013; Maleita et al., 2023).

Nematode management currently relies on a combination of approaches, including resistant cultivars, intercropping, crop rotation, biological control, and chemical nematicides. Synthetic nematicides can provide rapid and effective suppression and therefore remain important tools in intensive crop production. However, their repeated and intensive use can impose environmental and toxicological risks and may negatively affect non-target organisms and soil biological communities (Varandas et al., 2020). Moreover, the sustained use of nematicides has been associated with the development of resistances in some nematode populations, further undermining their long-term efficacy and reinforcing the need for diversified management approaches (Cabrera-Hidalgo et al., 2023; Abd-Elgawad, 2021).

The chemical toolbox available to growers has also progressively narrowed. The phase-out of broad-spectrum fumigants such as methyl bromide under the Montreal Protocol, together with the progressive restriction or deregistration of compounds including organophosphates such as fenamiphos, and carbamates such as carbofuran and aldicarb in the European Union and other agricultural regions has increased the pressure to develop alternative management strategies (Oka, 2020; Desaeger et al., 2020). European pesticide-reduction policies have further reinforced this transition. At the same time, however, the development of new nematicidal chemistries remains active, with several compounds displaying greater target specificity and potentially improved environmental profiles compared with older products (Desaeger et al., 2020). Thus, chemical control is unlikely to disappear in the short term but is increasingly expected to operate within more selective and integrated management programs.

Because no individual control method is consistently effective across nematode species, crops, soils, and climatic conditions, Integrated Nematode Management (INM) has emerged as the most appropriate framework for sustainable suppression. INM combines preventive cultural practices, host resistance, biological control agents, and were necessary, judicious application of chemical nematicides, with the relative contribution of each component adapted to local epidemiological and agronomic conditions (Sikora et al., 2023; Maleita et al., 2023). The objective is therefore not simply to eradicate PPN populations, which is rarely achievable or desirable, but to maintain nematode densities below economically damaging thresholds while preserving soil health and ecosystem functioning. Within this framework, biological control has gained increasing attention as a means of reducing reliance on synthetic nematicides. Microbial-based products containing *Bacillus* spp., *Purpureocillium lilacinum*, *Trichoderma* spp., and *Pochonia chlamydosporia* have demonstrated nematicidal or nematode-suppressive activity and have contributed to the expansion of the commercial bionematicide sector (Desaeger et al., 2020; Varandas et al., 2020; Ayaz et al., 2024). Nevertheless, the efficacy of microbial biocontrol agents can be highly context-dependent and may vary with soil properties, environmental conditions, crop genotype, microbial establishment, and interactions with the resident microbiome. These limitations highlight the need to move beyond approaches based solely on direct antagonism and to exploit microbial interactions that enhance the plant’s own capacity to resist nematode infection.

### Emergence of endophytes as modulators of plant defense

In recent years, endophytic microorganisms, including bacteria and fungi, have emerged as promising components of this next generation of biological control strategies. Endophytes colonize internal plant tissues without causing disease and can establish persistent interactions with their hosts. Rather than acting exclusively through direct antagonism of pathogens or pests, endophytes can modulate defense signaling pathways and reprogram host gene expression, thereby modifying the plant’s capacity to respond to subsequent biotic stress (Busby et al., 2016; Waqar et al., 2024). This interaction often involves the activation of induced systemic resistance (ISR) and the regulation of phytohormonal pathways such as jasmonic acid, ethylene, and salicylic acid signaling. Consequently, it contributes to improved plant resilience against biotic stress while maintaining host fitness (Gore et al., 2025).

## Endophytic microorganisms

Endophytes were historically considered as microorganisms that colonize internal plant tissues without causing apparent disease (Hardoim et al., 2015). However, a more recent definition explicitly incorporates the dynamic nature of endophytism, acknowledging that the same microorganism can switch between endophytic, pathogenic, or saprobic lifestyles depending on the plant’s growth stage or environmental stress. Such a functional perspective emphasizes that endophytism represents a continuum of plant-microbe interactions rather than a strictly defined ecological category and helps reconcile the classification of microorganisms that can behave as latent pathogens under specific conditions (Liao et al., 2025).

In this review, we use the term *endophytic fungi* to refer primarily to fungi capable of colonizing internal plant tissues without causing apparent disease, while recognizing that endophytism is a context-dependent and dynamic lifestyle. Our focus is on fungal taxa for which an endophytic or root-colonizing phase is relevant to their interaction with the plant and/or to the suppression of plant-parasitic nematodes. However, we also discuss selected rhizosphere-associated and nematophagous fungi when their activities provide relevant mechanistic context for direct nematode antagonism or complement plant-mediated protection. Importantly, these terms are not interchangeable: rhizosphere-associated refers to an ecological association with the root-soil interface, whereas nematophagous describes a functional capacity to antagonize nematodes through parasitism, predation, trapping, or other direct mechanisms. Thus, not all rhizosphere-associated or nematophagous fungi are considered endophytes in this review.

Endophytic communities comprise taxonomically diverse bacteria and fungi (Hardoim et al., 2015). Bacterial endophytes are predominantly affiliated with Proteobacteria (54%, including *Pseudomonas* and *Rhizobium*), Actinobacteria (20%, including antibiotic-producing *Streptomyces*), Firmicutes (16%, including insecticidal *Bacillus*), and Bacteroidetes (6%), whereas fungal endophytes are mainly represented by Glomeromycota (40%, arbuscular mycorrhizal fungi), Ascomycota (31%, including *Alternaria* and *Epichloë*), Basidiomycota (20%, including ectomycorrhizal symbionts), and Zygomycota (0.1%) (Hardoim et al., 2015). Importantly, bacterial and fungal endophytes cannot be clearly assigned to specific functions based solely on taxonomy. Closely related strains may range from beneficial to neutral or pathogenic depending on the host genotype and environmental context (Hardoim et al., 2015).

Endophytic microorganisms can enhance plant resilience through interconnected effects on nutrient acquisition, growth regulation, stress tolerance, and immunity. Root associated bacterial and fungal endophytes can contribute to the mobilization or acquisition of nutrients such as nitrogen, phosphorus, and iron, often through nitrogen fixation, phosphate solubilization, siderophore production, and the release of organic acids, and extracellular enzymes (Hardoim et al., 2015). They can also modulate plant development through the production or regulation of phytohormones, including auxins (indole-3-acetic acid), gibberellins, cytokinins, and abscisic acid, thereby promoting root development, shoot growth, and stress tolerance (Younas and Younas, 2025). At the same time, endophytic colonization can alter the plant immune state by activating or priming defense pathways involving jasmonic acid and ethylene signaling resulting in enhanced induced systemic resistance (ISR), preparing the plant to respond more rapidly and effectively to subsequent pathogen or nematode attacks (Pieterse et al., 2014; Fontana et al., 2021). These functions are not mutually exclusive and may act synergistically to increase plant resilience to both biotic and abiotic stresses.

For PPN management, fungal endophytes are particularly promising because they can combine direct antagonism of nematodes with indirect enhancement of host resistance. Some endophytic fungi produce secondary metabolites with nematocidal or nematostatic activity, which can affect nematode eggs, juveniles, or adults. In parallel, endophytic colonization can prime host plant defense responses, through ISR via jasmonic acid and ethylene signaling pathways (Mwangi et al., 2024; Vu et al., 2006). This dual mode of action distinguishes endophytic fungi from control strategies relying exclusively on direct toxicity and highlights their potential as components of sustainable agriculture, providing an effective, environmentally compatible, and economically viable strategy to combat nematode-induced crop losses while reducing dependence on hazardous synthetic pesticides.

## Endophytic fungi: from symbiosis to biocontrol

### Colonization and defense reprogramming by endophytic fungi

Endophytic fungi establish symbiotic associations with plants through a sophisticated process involving initial colonization, evasion of host immunity, and subsequent modulation of plant defense pathways. Rather than being passive inhabitants, endophytes engage in dynamic interactions in which fungal colonization and plant immune responses are continuously balanced to maintain compatibility.

The establishment of endophytic fungi within their host plants is a multistep process that begins in the surrounding environment. It is initiated when soil fungi recognize and responds to chemical signals, including sugars, organic acids, and other metabolites released by plant roots (Ray et al., 2018, as cited in Yan et al., 2019). Following this chemical communication, endophytic fungi are primarily recruited from the rhizosphere and gain access to the plant through the roots. They attach to the root surface and, in some cases, form specialized structures such as appressoria that facilitate penetration of the outer cell layers (Yedidia et al., 1999). Microscopic observations of early *Trichoderma* spp. colonization in tomato roots have shown that cell integrity is often maintained during this process, highlighting an important distinction from pathogenic invasions (Chacón et al., 2007). Colonization may also occur through two majors transmission routes: vertical transmission, in which fungi are transferred from the mother plant to progeny through the seed, and horizontal transmission, in which fungal propagules are dispersed between plants by wind, rain, insects or other vectors (Yan et al., 2019).

To survive and establish within host tissues, endophytic fungi have evolved diverse strategies to evade or counteract the plant’s first line of defense. One key mechanism involves controlling the oxidative burst generated by plants, which produces ROS as part of the defense response. For instance, in the root endophyte *Piriformospora indica*, the continuous release of low levels of ROS can stimulate the plant’s antioxidant machinery, including catalase and superoxide dismutase, thereby preconditioning the host and limiting the occurrence of a subsequent hypersensitive response (Fontana et al., 2021).

In addition, successful penetration and systemic colonization of plant tissues require appropriate enzymatic activities. Some beneficial microorganisms produce cell-wall-degrading enzymes that facilitate tissue entry without inducing strong pathogenic responses. For example, *Azoarcus* sp. produces endoglucanase, which facilitates entry without triggering pathogenic responses (Mengistu, 2020). Other endophytes may employ stealth strategies that minimize their recognition by the host immune system. Together, these mechanisms allow endophytic fungi to establish a compatible association while avoiding excessive activation of plant defenses.

Once established, endophytic fungi can actively reprogram the plant immune system, creating a balanced mutualistic interaction while priming the plant for enhanced protection against subsequent attacks. This phenomenon is commonly associated with ISR, which frequently involve jasmonic acid (JA) and ethylene (ET) signaling, in contrast to systemic acquired resistance (SAR), which is more typically associated with pathogens infection and salicylic acid (SA) signaling (Fontana et al., 2021; Mengistu, 2020). For instance, the root endophyte *P. indica* has been shown to induce resistance in barley against the fungal pathogen *Blumeria graminis* and in bananas against *Fusarium oxysporum f.* sp. *Cubense*, partly through activation of defense signaling and increased antioxidant enzyme activities (Fontana et al., 2021). Similarly, *Trichoderma* species are well-known for their ability to induce systemic resistance. For example, *T. harzianum* can trigger ISR in tomato against pathogens such as *Alternaria solani* and *Botrytis cinerea* through modulation of JA/ET signaling (Fontana et al., 2021). This priming enables plants to respond more rapidly and strongly when subsequently challenged by pathogens.

Endophytes can also strengthen both physical and chemical barriers. They can induce structural changes in the plant cell wall, including deposition of callose, pectin, and lignin, thereby creating a more effective physical barrier against invading organisms. In parallel, endophytic fungi can induce defense-related enzymes such as peroxidases, chitinases, and polyphenol oxidase (PPO), which can directly inhibit pathogen growth (Mengistu, 2020). *Trichoderma arundinaceum*, for example, produces trichodiene, a volatile compound that primes tomato defense genes associated with both SA- and JA-dependent pathways, thereby enhancing resistance (Malmierca et al., 2015). Beyond their roles in defense signaling, endophytes themselves can also function as active components of plant protection. Following pathogen perception, some endophytes, such as *Paraconiothyrium* strain SSM001 in yew, can migrate toward the site of attack and produce antifungal compounds, including taxol, thereby contributing to a localized extracellular chemical barrier (Soliman et al., 2015).

Overall, endophytic colonization involves a multifaceted reprogramming of plant defense, combining immune modulation, hormonal priming, structural reinforcement, and, in some cases, direct chemical antagonism. This interaction places the plant in a state of heightened defensive readiness while maintaining compatibility with the beneficial fungus. Such mechanisms make endophytic fungi particularly promising candidates for the biological control of plant-parasitic nematodes. Primed defense responses, especially those involving JA and ET signaling can enhance plant resistance to nematode invasion, interfere with feeding-site establishment, and ultimately reduce nematode reproduction and survival (Rodriguez et al., 2009; Fontana et al., 2021).

### Biological control of nematodes by endophytic fungi

Endophytic fungi with nematicidal potential have emerged as promising agents for the biological control of plant parasitic nematodes (Poveda et al., 2020; Sikora et al., 2008). Their activity can be mediated through two main, non-exclusive strategies: direct antagonistic mechanisms and indirect, plant-mediated mechanisms (**Figure 3**). Direct mechanisms involve the infection, capture, or intoxication of nematodes by the fungus itself, and include egg parasitism, predation or trapping, direct nematicidal activity, and the production of compounds that impair nematode survival or development, all of which affect the nematode independently of the activation of host defenses. Indirect mechanisms, by contrast, rely primarily on the fungal reprogramming of plant defense responses, whereby fungal colonization or fungal-derived signals modify plant responses and thereby reduce nematode infection, reproduction, or damage. Distinguishing these mechanisms is important because the presence of a fungal metabolite or an interaction with a nematode does not necessarily imply activation of plant immunity.

**Figure 3:**
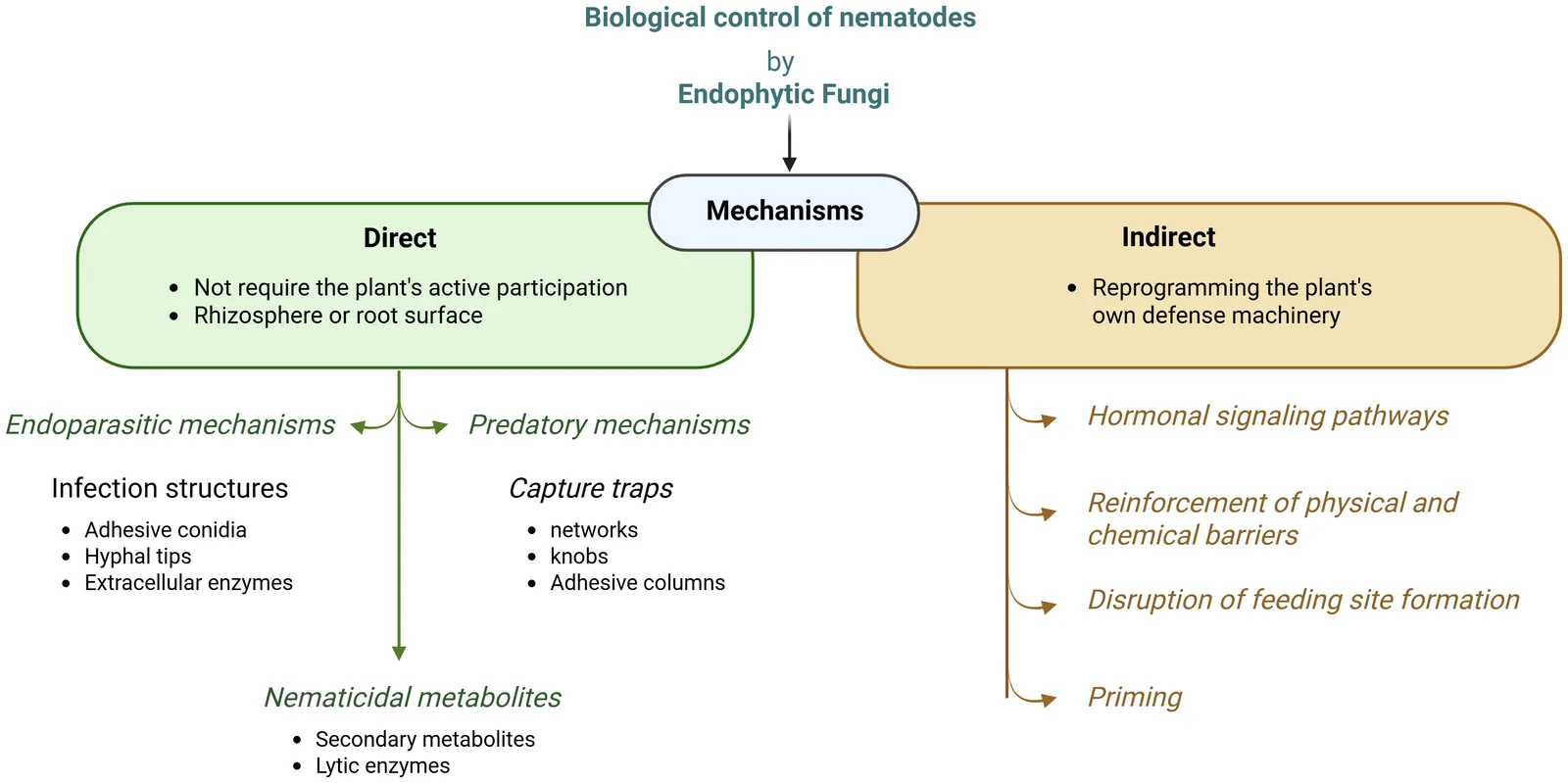
Mechanisms of nematode control by endophytic fungi. Endophytic fungi can suppress plant-parasitic nematodes through both direct and indirect mechanisms. Direct mechanisms are largely independent of host plant activity and occur in the rhizosphere or at the root surface. They include endoparasitic mechanisms mediated by infection structures such as adhesive conidia and hyphal tips, together with extracellular lytic enzymes; predatory mechanisms involving specialized trapping structures, including adhesive networks, knobs, and columns; and the production of nematicidal compounds, including secondary metabolites and lytic enzymes. Indirect mechanisms involve reprogramming of the plant’s defense machinery through activation of hormone-mediated signaling pathways, reinforcement of physical and chemical barriers, interference with nematode feeding-site formation, and priming of systemic plant defense responses. Created in https://BioRender.com.

### Direct antagonism against nematodes

Some fungi associated with plants can exert direct antagonistic effects on nematodes through parasitism, predation, trapping, or the production of nematicidal compounds. These mechanisms are not restricted to endophytic fungi and may also occur in specialized nematophagous fungi that primarily occupy the rhizosphere or soil environment. In this context, nematophagous fungi are considered here as a complementary functional group rather than as a synonym for endophytic fungi. Their inclusion is relevant because direct nematode antagonism can coexist with, or complement, plant-mediated protection associated with root-colonizing and endophytic fungi.

*Parasitism and capture structures.* Nematophagous fungi have evolved a remarkable diversity of specialized structures that enable them to recognize, infect, capture and kill nematodes. These mechanisms are broadly divided into two major categories: endoparasitic mechanisms, involving specialized infection structures and predatory mechanisms, involving specialized trapping devices (Moosavi and Zare, 2020).

*Endoparasitic mechanisms: infection structures.* Endoparasitic fungi, including *Pochonia chlamydosporia* and *Purpureocillium lilacinum*, can infect nematode eggs and females through specialized infection structures. *P. chlamydosporia* produces adhesive conidia and hyphal tips that attach to the cuticle of nematode eggs and females. Following attachment, the fungus secretes a battery of extracellular hydrolytic enzymes, including proteases, chitinases, and collagenases, which degrade the eggshell and cuticle, thereby facilitating hyphal penetration and internal colonization (Lopez-Llorca et al., 2002; Manzanilla-López et al., 2013). Recent proteomic studies indicate that *P. chlamydosporia* employs a highly coordinated molecular strategy during infection. At early stages, the fungus rapidly adjusts its metabolism and protein synthesis while simultaneously enhancing antioxidant defenses to cope with the host defense environment and associated stress. At later stages, metabolic pathways are further modulated, and DNA replication is enhanced to support fungal proliferation (Ma et al., 2025). A diverse repertoire of transmembrane proteins also appears to contribute to host recognition, adhesion, and the formation of invasive structures (Ma et al., 2025). Similarly, *Purpureocillium lilacinum* (syn. *Paecilomyces lilacinus*) efficiently parasitizes eggs of *Meloidogyne* spp. through the production of extracellular proteases and chitinases that compromise eggshell integrity and reduce egg viability (Khan et al., 2006; Sharma et al., 2021). Genomic analyses have revealed that *P. lilacinum* possesses an extensive repertoire of genes encoding G-protein coupled receptors (GPCRs), proteases, glycoside hydrolases, and carbohydrate esterases, which may contribute to host recognition and eggshell degradation together with genes involved in the biosynthesis of diverse secondary metabolites (Zhang et al., 2024). Recent studies reported egg and female mortality rates of 74.33% and 92.5%, respectively, under laboratory conditions, while field application reduced mobile nematode populations by 57.7% within 15 days (Nguyen Thi et al., 2025). The production of chitinases and proteases is central to the degradation of nematode eggshells and cuticles, and fungal efficacy can be further enhanced by nitrogen-rich organic substrates, which increase protease activity and egg-hatching inhibition up to 96.8% (Sharma et al., 2021).

*Predatory mechanisms: capture traps.* Predatory fungi, particularly members of the family Orbiliaceae (order Orbiliales), have evolved highly sophisticated hyphal structures that enable them to capture and kill motile nematodes. Based on the morphology of their trapping devices, these fungi can be classified into four main types (Moosavi and Zare, 2020; Yang et al., 2012) (see **Table 1**).

**Table 1:** Classification of predatory fungi from the family Orbiliaceae based on trap type (adapted from Moosavi and Zare, 2020).

Trap Type	Mechanism	Representative Genera
Adhesive networks	The most common trap type; hyphal outgrowths form three-dimensional adhesive loops that entangle nematodes	*Arthrobotrys*
Constricting rings	Rings of three cells that rapidly swell inward (in 1-2 seconds) upon contact, "lassoing" the nematode	*Drechslerella*
Adhesive knobs	Stalked or unstalked spherical adhesive cells that capture nematodes upon contact	*Dactylellina*
Adhesive columns	Layers of adhesive cells on hyphae that trap nematodes	*Gamsylella*

Adhesive traps, including networks, knobs, and columns, contain dense cytoplasmic inclusions and abundant rough- and smooth-surfaced membranes that are largely absent from vegetative hyphae. These features are thought to be associated with the synthesis and secretion of adhesive substances. In contrast, constricting rings exhibit a distinctive ultrastructure that changes dramatically upon closure. In the open state, they contain membrane-bound inclusions and labyrinthine networks along the luminal side surface, whereas these structures disappear following closure and the cytoplasm becomes less densely stained (Yang et al., 2012). Constricting rings represent a remarkable mechanical adaptation that enables rapid nematode capture without relying on the continuous toxin production (Moosavi and Zare, 2020).

Predatory nematophagous fungi are widely distributed, with a higher prevalence in temperate regions, and are commonly encountered in soil and wood-associated habitats (Moosavi and Zare, 2020). Their trapping strategies are considered a striking example of convergent evolution among mycelial fungi inhabiting nitrogen-limited environments, where nematode predation provides and additional source of nitrogen to support fungal growth and reproduction (Moosavi and Zare, 2020). Recent reviews further emphasize the functional diversity of nematophagous fungi, encompassing trapping, egg and female parasitism, toxin production, and other mechanisms that contribute to direct nematode suppression in soil (Ciancio and Picciotti, 2026).

*Production of nematicidal metabolites.* Many endophytic fungi secrete secondary metabolites and lytic enzymes that can directly kill or immobilize nematodes (**Table 2**). *Trichoderma* spp., for instance, produce peptaibols and other nematicidal compounds that can disrupt nematode membranes or interfere with their nervous system (Zhang et al., 2020). These fungi also release extracellular enzymes including proteases, chitinases, and collagenases that degrade the nematode cuticle and eggshell, thereby reducing egg hatching and juvenile viability (Lopez-Llorca et al., 2002; Manzanilla-López et al., 2013; Hastuti et al., 2022). Recent studies further support the direct nematicidal activity of metabolites released by endophytic fungi into culture filtrates. For example, culture filtrates from the endophytic fungi *Penicillium citrinum*, *Aspergillus niger*, and *A. fumigatus* reduced the viability of *Meloidogyne javanica* juveniles and inhibited egg hatching, with an 8% filtrate of *P. citrinum* showing particularly strong activity after 24 h. GC–MS analysis identified 22 compounds in the fungal filtrates, including quinazoline derivatives and fatty acids such as palmitic acid, providing candidate metabolites associated with the observed nematicidal activity (Baazeem et al., 2022). Similarly, culture filtrates from several endophytic fungi, including *Fusarium solani*, *Cephalosporium* sp., *Curvularia* spp., and *Talaromyces* spp., exhibited nematicidal activity against *M. javanica*; among the fungi tested, *F. solani* caused 100% juvenile mortality after 48 h, and GC–MS analysis of its culture filtrate revealed a diversity of fungal metabolites (Farhat et al., 2022). More recently, *Aspergillus flavus*, an endophyte isolated from *Trigonella foenum-graecum* seeds, produced a range of secondary metabolites, including gadoleic acid, oleic acid, and palmitic acid, in culture filtrates that inhibited egg hatching and increased juvenile mortality of *Meloidogyne incognita*. Under greenhouse conditions, application of the fungal filtrate reduced gall formation and egg masses by 90% and 89.5%, respectively, while also increasing the activity of defense-related enzymes in potato plants (Ghareeb et al., 2024).

**Table 2:** Nematicidal metabolites of endophytic fungi: chemical diversity, activity spectra, and modes of action.

Metabolite	Chemical Class	Fungal Source	Endophytic Host	Target Nematode	Key Activity	Mechanism of Action	Reference(s)
Guignardone D	Dioxalanone	*Guignardia mangiferae*	*Persea indica*	*Meloidogyne javanica*	Strong nematicidal activity	Not fully characterized; proposed membrane disruption or interference with cuticle integrity	Andrés et al. (2017)
Chaetoglobosin A	Cytochalasan alkaloid	*Chaetomium globosum*	*Caesalpinia sepiaria*, *Cupressus* sp.	*Meloidogyne incognita*, *M. javanica*	LC50 = 77.0 µg/mL against J2 juveniles (72 h); high mortality at 200 µg/mL	Actin cytoskeleton disruption; inhibits actin polymerization, leading to impaired motility and feeding	Hu et al. (2013); Khan et al. (2019)
Chaetoglobosin B	Cytochalasan alkaloid	*Chaetomium globosum*	*Caesalpinia sepiaria*	*Meloidogyne incognita*, *M. javanica*	Moderate nematicidal activity; high mortality at 200 µg/mL	Actin cytoskeleton disruption (similar to chaetoglobosin A)	Khan et al. (2019)
Flavipin	Polyketide	*Chaetomium globosum*	*Caesalpinia sepiaria*	*Meloidogyne javanica*	High mortality rates at 200 µg/mL	Induces oxidative stress; disrupts cellular redox homeostasis	Khan et al. (2019)
3-Methoxyepicoccene	Terpenoid	*Chaetomium globosum*	*Caesalpinia sepiaria*	*Meloidogyne javanica*	Moderate nematicidal activity	Not fully characterized; potential membrane disruption	Khan et al. (2019)
4,5,6-Trihydroxy-7-methylphthalide	Phthalide derivative	*Chaetomium globosum*	*Caesalpinia sepiaria*	*Meloidogyne javanica*	Moderate nematicidal activity	Not fully characterized	Khan et al. (2019)
Ergovaline	Ergot alkaloid	*Epichloë* spp. (e.g., *E. coenophialum*)	*Festuca arundinacea* (tall fescue)	*Pratylenchus scribneri*, various PPN	Active at 1–5 µg/mL *in vitro*; repellent and toxic	Neurotoxicity; acts as a dopamine receptor agonist, disrupting neuromuscular coordination and feeding behavior	Bacetty et al. (2009a, 2009b)
2-Butanone	Volatile organic compound (VOC)	*Induratia coffeana*, *Induratia* sp.	*Coffea arabica* (coffee)	*Meloidogyne incognita*	Reduces infectivity; causes juvenile mortality	Chemotaxis disruption; interferes with nematode host location and orientation; neurotoxic effects	da Silva Costa Guimarães et al. (2021)
4-Hydroxybenzoic acid	Phenolic acid	*Fusarium oxysporum* (non-pathogenic strain Fo162)	*Solanum lycopersicum* (tomato)	*Meloidogyne incognita*	LC50 = 104 µg/mL	Oxidative stress induction; disrupts cell membrane integrity	Bogner et al. (2017)
Gibepyrone D	Pyrone derivative	*Fusarium oxysporum* (non-pathogenic strain Fo162)	*Solanum lycopersicum* (tomato)	*Meloidogyne incognita*	LC50 = 134 µg/mL	Not fully characterized; potential interference with nematode development	Bogner et al. (2017)
Indole-3-acetic acid (IAA)	Auxin phytohormone	*Fusarium oxysporum* (non-pathogenic strain Fo162)	*Solanum lycopersicum* (tomato)	*Meloidogyne incognita*	LC50 = 117 µg/mL	Hormonal disruption; interferes with nematode feeding site establishment and root penetration	Bogner et al. (2017)

J2, second-stage juvenile; LC_50_, median lethal concentration; PPN, plant-parasitic nematodes; VOC, volatile organic compound.

However, relatively few individual metabolites have been structurally characterized and their mode of action experimentally validated. Among the most promising characterized nematicidal compounds, Guignardone D (also referred to as Guignardianone D in some sources) is of particular interest. This compound was identified during a biodiversity study of endophytic fungi associated with the Macaronesian laurel forest (laurisilva), a unique and ancient ecosystem. It was isolated from *Guignardia mangiferae*, an endophyte of *Persea indica*, a tree species endemic to the Canary Islands and Madeira (Andrés et al., 2017). Chemically, Guignardone belongs to the relatively uncommon class of dioxalanone, and exhibit strong nematicidal activity against *Meloidogyne javanica*, one of the most economically damaging PPN worldwide. Although its precise mode of action remains to be fully elucidated, available evidence suggests that its activity may involve disruption of cellular or cuticular structures (Andrés et al., 2017; Rojas López-Menchero et al., 2026). The discovery of Guignardone D highlights the potential of exploring underexplored ecological niches for the identification of novel fungal-derived nematicidal compounds.

Beyond Guignardone D, several other secondary metabolites produced by endophytic fungi have been characterized for their nematicidal properties. The endophytic fungus *Chaetomium globosum*, isolated from plants such as *Caesalpinia sepiaria* and *Cupressus* sp., is a prolific producer of nematicidal metabolites. This fungus produces a diverse cocktail of bioactive compounds, including the cytochalasan alkaloids chaetoglobosin A and chaetoglobosin B, as well as flavipin, 3-methoxyepicoccone, and 4,5,6-trihydroxy-7-methylphthalide (Khan et al., 2019; Rojas López-Menchero et al., 2026). Chaetoglobosin A exhibits particularly strong activity, with a median lethal concentration (LC_50_) of 77.0 µg/mL against second-stage juveniles (J2) of *Meloidogyne incognita* after 72 h of exposure (Hu et al., 2013). Its mode of action involves disruption of the actin cytoskeleton through inhibition of actin polymerization, resulting in impaired nematode motility and feeding and ultimately leading to mortality (Hu et al., 2013). Chaetoglobosin B appears to act through a similar mechanism, although with lower activity. Flavipin, a polyketide, has been reported to induce oxidative stress by disrupting cellular redox homeostasis, thereby contributing to high mortality in *M. javanica* (Khan et al., 2019). The simultaneous production of multiple metabolites with distinct biological activities may contribute to overall nematicidal efficacy of *C. globosum*, although the extent to which these compounds act synergistically remains to be determined.

Ergovaline, an ergot alkaloid produced by *Epichloë* endophytes, including *E. coenophialum*, during colonization of grasses such as tall fescue (*Festuca arundinacea*), provides another example of an endophyte-derived nematicidal metabolite. *In vitro* assays have demonstrated activity at remarkably low concentrations, ranging from 1 to 5 µg/mL against *Pratylenchus scribneri* and other PPNs (Bacetty et al., 2009a, 2009b). Ergovaline acts as a neurotoxin compound, and can interfere with neurotransmitter signaling, thereby disrupting neuromuscular coordination and feeding behavior in nematodes (Bacetty et al., 2009b). This compound illustrates the broader ecological role of endophyte-derived alkaloids in protecting host plants against herbivores and nematodes through both antifeedant and direct toxic mechanisms.

Recent evidence further supports the nematicidal potential of root-associated fungal endophytes against migratory nematodes. Rodríguez-Zamora et al. (2026) isolated endophytic fungi from plantain roots and found that isolates of *Fusarium* and *Trichoderma* caused 58.8% and 51.6% mortality, respectively, of *Radopholus similis* after 24 h, compared with 8.1% in the control. These results expand the evidence for direct nematicidal activity of endophytic fungi beyond root-knot nematodes.

Volatile organic compounds (VOCs) also contribute to long-distance interactions between fungi and nematodes in the rhizosphere. 2-Butanone, produced by *Induratia* species, including *I. coffeana* isolated from coffee plants (*Coffea arabica*), has been shown to reduce the infectivity of *M. Incognita* juveniles and induce direct mortality (da Silva Costa Guimarães et al., 2021). As a VOC, 2-butanone may act before physical contact by modifying nematode behaviour and chemotaxis potentially interfering with the ability of juveniles to locate and penetrate host roots, representing a unique mode of action distinct from contact toxins (Rojas López-Menchero et al., 2026). Other studies further illustrate the diversity of fungal VOCs with direct nematicidal activity. VOCs emitted by the endophytic fungus *Daldinia cf. concentrica*, as well as a synthetic mixture containing 3-methyl-1-butanol, 2-methyl-1-butanol, 4-heptanone, and isoamyl acetate, reduced *M*. *javanica* J2 viability and egg hatching and suppressed root galling and egg production in tomato (Liarzi et al., 2016). Similarly, the nematophagous fungus *Duddingtonia flagrans* combines nematode trapping through adhesive networks with the production of toxic VOCs, including cyclohexanamine, cyclohexanone, and cyclohexanol, which exhibit nematicidal activity against *M. incognita* juveniles and inhibit egg hatching (Mei et al., 2021). These findings complement the nematicidal and nematostatic effects reported for VOCs produced by *Muscodor albus* against several plant-parasitic nematodes (Riga et al., 2008), highlighting VOC-mediated toxicity as a direct mechanism that can operate without physical contact between fungal structures and nematodes. More recently, Consoli et al. (2026) showed that a biorational mixture of VOCs derived from *Muscodor* spp. reduced the motility and survival of both *M*. *incognita* and *Pratylenchus neglectus*, while also reducing root galling and nematode root invasion in planta. These findings further support the potential of fungal VOCs as direct nematicidal agents and as candidates for biorational nematode management.

Finally, the non-pathogenic strain *Fusarium oxysporum* Fo162, isolated from tomato roots, has been extensively studied for its biocontrol potential against *M. incognita*. This endophyte produces a combination of metabolites, including 4-hydroxybenzoic acid, gibepyrone D, and the phytohormone indole-3-acetic acid (IAA), which have been associated with nematicidal activity (Bogner et al., 2017). Their respective LC_50_ values of 104 µg/mL, 134 µg/mL, and 117 µg/mL against *M. incognita* indicate moderate but significant activity. 4-Hydroxybenzoic acid may act through oxidative stress induction and membrane disruption, while IAA may interfere with hormonal signaling during nematode feeding site establishment (Bogner et al., 2017). This example highlights the potential of non-pathogenic endophytic fungi as sources of diverse bioactive compounds and underscores the possibility that some fungi may simultaneously contribute to plant growth promotion and nematode suppression.

#### Limitations of direct mechanisms

Despite their efficacy under controlled laboratory conditions, direct antagonistic mechanisms face several challenges *in planta*. Their effectiveness is strongly influenced by environmental factors, including temperature, pH, moisture and nutrient availability, as well as competition with native soil microbiota. Moreover, the transition from a saprophytic to a parasitic lifestyle may not occur efficiently when alternative carbon and nutrient sources are readily available, potentially reducing nematode capture or parasitism under field conditions (Rahman et al., 2024). Consequently, the strong nematicidal activity frequently observed under laboratory conditions does not necessarily translate into consistent efficacy in planta or in the field. Understanding how fungal establishment, metabolite production, and nematode interactions are modulated by the plant and soil environment will therefore be critical for translating these direct mechanisms into reliable biological control strategies.

### Indirect plant-mediated mechanisms: defense reprogramming by endophytic fungi

Beyond direct antagonism, endophytic fungi can protect plants against nematodes indirectly by reprogramming the plant’s own defense machinery. This reprogramming can involve the modulation of hormonal signaling, reinforcement of structural and chemical barriers, and interference with nematode feeding-site establishment, ultimately creating a less favourable environment for nematode infection and development.

#### Activation of hormonal signaling pathways

Endophytic colonization can prime the plant immune system, establishing a state of enhanced readiness known as ISR. Depending on the fungal species and plant–fungus combination, this response can involve JA and ET signaling pathways and their interactions with other hormonal pathways. For example, root colonization by *Trichoderma harzianum* in tomato has been shown to enhance the expression of JA-responsive genes following infection by *M. incognita*, resulting in reduced gall formation and nematode reproduction (Fontana et al., 2021; Poveda et al., 2020). Similarly, *Fusarium oxysporum* strain Fo162 was shown to induce ISR in tomato against *M. incognita* through modulation of JA/ET-associated defense responses, thereby reducing nematode penetration and delaying its development (Bogner et al., 2017). These findings illustrate how endophytic fungi can modify the hormonal balance of the host to enhance the speed and magnitude of defense activation following nematode attack. The importance of fungal establishment prior to nematode attack is further supported by recent work in olive, where preventive application of endophytic *T. harzianum* and *Bacillus subtilis*, particularly when applied in combination, resulted in greater suppression of *Meloidogyne javanica* and *M. incognita* than post-inoculation treatments. Preventive application reduced galling, egg production, and nematode reproduction while improving plant growth and attenuating oxidative damage (Laasli et al., 2026).

Interestingly, *Beauveria bassiana* provides an example in which direct and plant-mediated effects may coexist. In tomato, inoculation with this endophytic fungus reduced *M. incognita* root colonization and was associated with the induction of plant defense hormones, while its fermentation broth showed direct effects on egg hatching, J2 survival, and nematode repellence (Fang et al., 2025). These findings illustrate the difficulty of assigning fungal-mediated nematode suppression to a single mechanism when both fungal-derived compounds and host defense responses may contribute to the outcome.

#### Reinforcement of physical and chemical barriers

Endophytic fungi can also enhance constitutive or inducible structural defenses by stimulating modifications of the plant cell wall and the accumulation of defensive compounds. These responses may include deposition of callose, lignin, and phenolic compounds, thereby strengthening physical barriers to nematode penetration and migration. For instance, colonization of cucumber roots by *P. chlamydosporia* has been associated with increased lignification and enhanced activities of defense-related enzymes such as peroxidases and polyphenol oxidases, responses that can restrict *M. incognita* penetration and gall development (Escudero and Lopez-Llorca, 2012). In parallel, the accumulation of pathogenesis-related (PR) proteins and phytoalexins may further contribute to an unfavorable environment for nematode development (Mengistu, 2020).

#### Interference with nematode feeding site establishment

A particularly important indirect mechanism against sedentary endoparasitic nematodes, such as *Meloidogyne* spp. and *Heterodera* spp. is the disruption of specialized feeding-site formation. Endophytic colonization can alter the expression of plant genes involved in the differentiation and maintenance of giant cells or syncytia, thereby compromising the establishment of functional feeding-sites. For example, *F. oxysporum* Fo162 has been reported to downregulate auxin-responsive genes required for giant cell ontogeny, leading to smaller and less functional feeding sites (Bogner et al., 2017). Similarly, *Trichoderma* spp. can interfere with cellular processes associated with giant-cell development, including cell cycle regulation and endoreduplication, thereby impairing giant cell maturation (Poveda et al., 2020). Because these specialized feeding structures constitute the primary nutrient source for sedentary nematodes, their impaired development can directly restrict nematode growth, reproduction, and survival.

#### Priming as a cost-effective strategy

Importantly, fungal-mediated defense reprogramming does not necessarily involve constitutive activation of defense pathways. Instead, endophytic fungi can establish a primed state in which defense responses remain relatively low under non-challenged conditions but are activated more rapidly and strongly following nematode attack. Priming therefore provides an energy-efficient strategy that limits the fitness costs associated with maintaining high levels of constitutive defense (Conrath et al., 2015; Fontana et al., 2021). For instance, tomato plants colonized by *Trichoderma asperellum* can maintain defense-gene expression at levels comparable to those of non-colonized plants under basal conditions, while exhibiting a stronger induction of JA/ET-associated defense and oxidative response following *M. incognita* infection (Fontana et al., 2021). Thus, rather than continuously activating costly defense responses, the endophyte effectively increases the responsiveness—or preparedness—of the host immune system.

In summary, direct and indirect mechanisms represent complementary modes of fungal-mediated nematode suppression. Direct antagonistic mechanisms can provide immediate nematocidal activity through parasitism, predation or toxic metabolites, but its effectiveness may depend strongly on environmental conditions. In contrast, the indirect reprogramming of plant defenses can provide systemic and potentially longer-lasting protection by enhancing the plant’s capacity to detect and restrict nematode infection. The ability of some endophytic fungi to combine these direct and indirect modes of action makes them particularly versatile candidates for sustainable nematode management (Rahman et al., 2024) (**Figure 4**).

**Figure 4:**
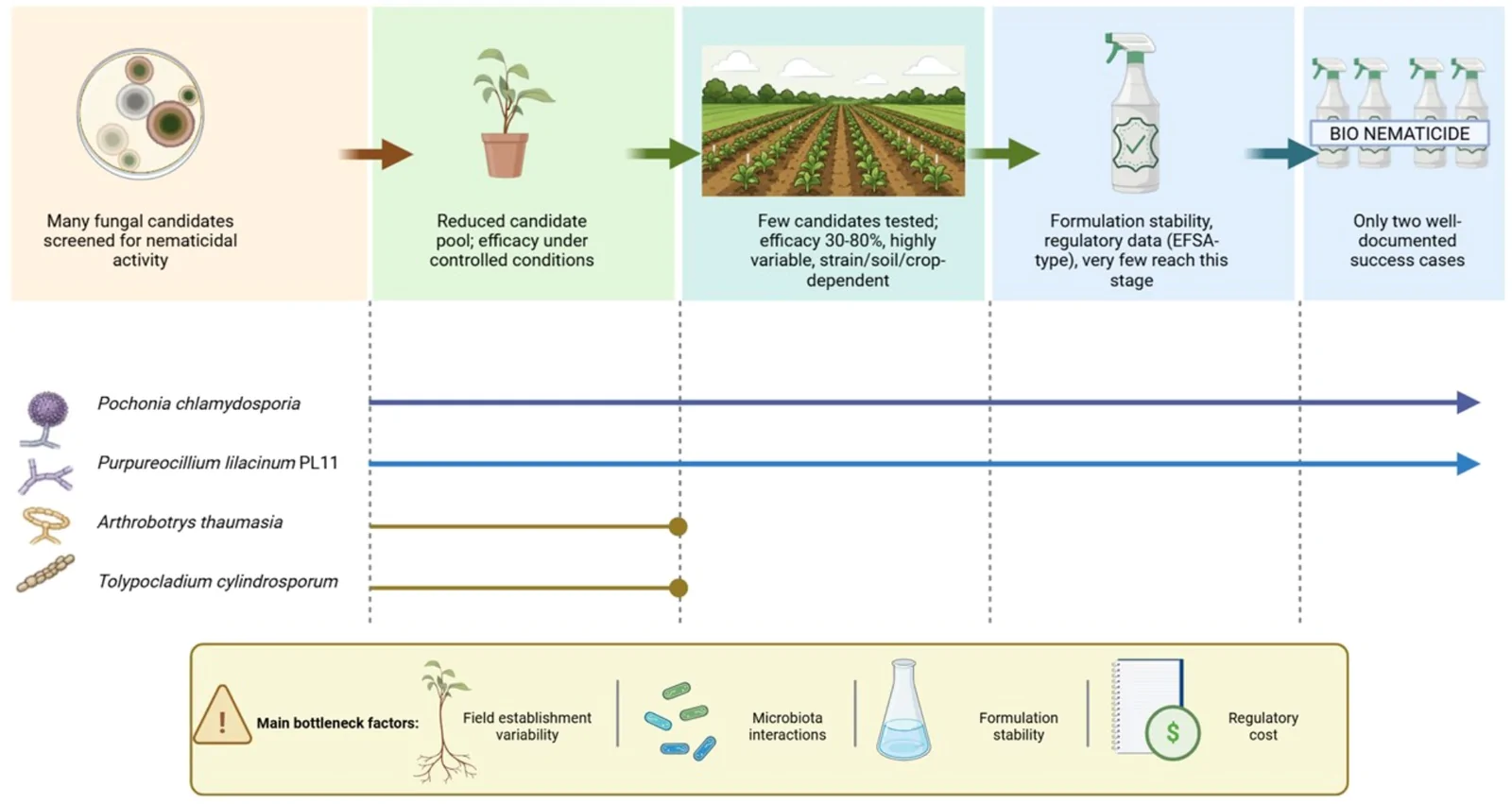
Endophyte-mediated restoration of nematode-suppressed plant immunity. Plant-parasitic nematode and endophytic fungi converge on shared host immune pathways, but exert opposing effects on their activitys. Left: nematode effectors secreted during root infection suppress key layers of plant defense, including pattern-triggered immunity (PTI), effector-triggered immunity (ETI, exemplified by NRC4 degradation), reactive oxygen species (ROS) production, callose deposition, and jasmonic acid/ethylene (JA/ET) signaling. Right: root colonization by endophytic fungi, including Trichoderma spp., Fusarium oxysporum Fo162, Pochonia chlamydosporia, primes these same defense pathways, enhancing their capacity for rapid activation upon subsequent nematode attack rather than constitutively activating defense responses. This antagonistic regulation of shared immune nodes illustrates a central molecular conflict in plant–nematode interactions: endophyte-induced priming can counteract nematode-mediated immune suppression and restore the host’s capacity to mount an effective defense response. Created in https://BioRender.com.

## From laboratory to field: challenges and opportunities

Although numerous endophytic fungi have demonstrated promising nematicidal activity under controlled laboratory conditions (Rojas López-Menchero et al., 2026), relatively few have progressed to successful greenhouse validation (Ghareeb et al., 2024), and only a limited number have reached registered commercial products. The commercialization of fungal bionematicides remains constrained by inconsistent field performance, economic considerations, formulation challenges, and regulatory requirements (Wilson and Jackson, 2013; Abd-Elgawad and Askary, 2020). This gap between proof-of-concept studies and practical implementation remains one of the major bottlenecks limiting the widespread adoption of fungal bionematicides. Importantly, single factors rarely determine the transition from laboratory efficacy to reliable field performance. Rather, it depends on complex interactions among the fungal strain, host plant, soil environment, native microbiota, formulation technology, application strategy, and agriculture practices.

Among the candidates evaluated to date, two species exemplify the relatively rare trajectory from laboratory research to commercial application: *Pochonia chlamydosporia* and *Purpureocillium lilacinum* (Ali et al., 2024; Ciancio et al., 2016; Sellitto et al., 2016). Both are primarily egg-parasitic, root-associated fungi that can also establish an endophytic phase within plant roots, and both have been evaluated beyond controlled pot experiments through field trials across multiple crops and geographic regions.

*Pochonia chlamydosporia* is among the most extensively characterized fungal biocontrol agents and has been developed into formulations targeting root-knot *(Meloidogyne* spp.) and cyst (*Globodera* spp.*, Heterodera* spp.) nematodes. Field trials against potato cyst nematodes in the UK provided an early example of rigorous, multi-season field validation (Tobin et al., 2008). However, efficacy has varied substantially among studies, with reduction in egg and juvenile populations typically ranging from approximately 30 to 80%, depending on the fungal isolate, application rate, crop, and prevailing soil conditions.

*Purpureocillium lilacinum*, meanwhile, provides an important example of regulatory translation. The European Food Safety Authority (EFSA) evaluated and supported the approval of strain PL11 as a microbial nematicide for use on eight vegetable crops in both field and greenhouse conditions (European Food Safety Authority, 2022). Such, regulatory recognition across multiple crops remains relatively uncommon among fungal bionematicides and illustrates the substantial progression required to move from demonstrated biological activity to an approved agricultural product. Beyond these two more advanced cases, several root-associated fungal candidates remain at earlier stages of development. Species such as *Arthrobotrys thaumasia* and *Tolypocladium cylindrosporum* have shown promising nematicidal activity in greenhouse experiments (Purba et al., 2022; Kassam et al., 2021). **Table 3** summarizes recent primary evidence (2021–2026) for these and other fungal biocontrol candidates, stratified by evidence level (*in vitro*, greenhouse/pot, and field); it shows that while *A. thaumasia* and *T. cylindrosporum* have not progressed beyond greenhouse/pot-level validation in any study identified for this period, genera with a longer development history, including *Pochonia chlamydosporia*, *Trichoderma* spp., and *Purpureocillium lilacinum*, have already reached field-level evidence, underscoring the developmental gap separating these two candidates from more advanced fungal bionematicides. In parallel, integrated biological management strategies combining complementary microbial agents are being explored to improve nematode suppression while supporting broader soil health. For example, field studies in Kenyan coffee production systems have evaluated combinations of *Trichoderma asperellum* and *Purpureocillium lilacinum* (Saikai et al., 2023). Whether these and other emerging candidates can progress from experimental efficacy to consistent field performance and ultimately commercial registration remains an important open question.

**Table 3:** Fungal biocontrol agents against plant-parasitic nematodes.

Fungus/strain	Host	Nematode	Mechanism	Experimental system	Outcome	Evidence level	Reference (s)
*Arthrobotrys thaumasia*	Tomato	*M. incognita, C. elegans*	*Trapping / culture filtrate toxicity*	In vitro (agar plate)	82% parasitism; 57.7% mortality	In vitro	Kassam et al. (2021)
*Tolypocladium cylindrosporum*	Tomato	*M. incognita, C. elegans*	*Culture filtrate toxicity*	In vitro (agar plate)	87.3% mortality (filtrate)	In vitro	Kassam et al. (2021)
*A. thaumasia*	Tomato	*M. incognita*	*Trapping*	In vitro (J2 exposure)	72.5–77.5% J2 reduction	In vitro	Eken et al. (2024)
*A. thaumasius*	Banana	*Meloidogyne spp.*	*Trapping / attraction*	In vitro (split-plate assay)	>65% attraction/capture (3 of 4 isolates)	In vitro	Carpio et al. (2026)
*Duddingtonia flagrans*	—	*M. incognita*	*Trapping + volatile toxicity (cyclohexanamine)*	In vitro (SEM, GC-MS, mortality assay)	97.9% mortality at 8.71 µM	In vitro	Mei et al. (2021)
*Fusarium solani* (endophyte)	—	*M. javanica*	*Culture filtrate toxicity*	In vitro + GC-MS profiling	Significant nematicidal activity	In vitro	Farhat et al. (2022)
*Aspergillus flavus* (endophyte)	— (isolated from fenugreek)	*M. incognita*	*Culture filtrate toxicity*	In vitro (filtered broth) + GC-MS	Nematicidal metabolites identified (gadoleic, oleic, palmitic acids)	In vitro	Ghareeb et al. (2024)
*Penicillium citrinum, Aspergillus niger, A. fumigatus* (endophytes)	— (isolated from Taify roses)	*M. javanica*	*Culture filtrate toxicity*	In vitro (mortality assay)	Up to 73% J2 mortality	In vitro	Baazeem et al. (2022)
*Endophytic fungi* (unspecified spp.)	Plantain roots	*Radopholus similis*	*Metabolite toxicity*	In vitro	Nematicidal activity confirmed	In vitro	Rodríguez-Zamora et al. (2026)
*A. thaumasia, A. musiformis*	Tomato	*M. hapla*	*Trapping*	Pot trial (CRD)	Significant J2 reduction (isolate-dependent)	Greenhouse/pot	Purba et al. (2022)
*A. thaumasia, T. cylindrosporum*	Tomato	*M. incognita*	*Trapping + parasitism*	Greenhouse (pot)	Reduced galling/reproduction (species-dependent)	Greenhouse/pot	Kassam et al. (2021)
*Pochonia chlamydosporia + Pasteuria penetrans*	Tomato	*M. incognita*	*Egg parasitism + combined BCA*	Greenhouse, 3 soil types	Up to 87% J2 reduction (combined)	Greenhouse/pot	Loffredo et al. (2024)
*Beauveria bassiana*	Tomato	*M. incognita*	*Repellent VOCs + endophytic colonization*	In vitro + in vivo (pot)	Strong J2 repellence; reduced infection in planta	Greenhouse/pot	Fang et al. (2025)
*Trichoderma harzianum + Bacillus subtilis*	Olive	*M. javanica, M. incognita*	*Endophytic antagonism; preventive vs curative timing*	Greenhouse	Nematode suppression; host biochemical response	Greenhouse/pot	Laasli et al. (2026)
*Muscodor spp.* VOCs (Formula B)	Cucumber	*M. incognita, Pratylenchus neglectus*	*Volatile nematicidal compounds*	In vitro + in vivo (pot)	90% mortality at 6h; reduced gall formation	Greenhouse/pot	Consoli et al. (2026)
*Purpureocillium lilacinum* + PGPR bacteria	Tomato	*M. incognita*	*Filtrate toxicity + growth promotion*	Greenhouse (in vivo)	Reduced egg hatching; improved plant growth	Greenhouse/pot	Ali et al. (2024)
*P. lilacinum, Pochonia chlamydosporia*	Common bean (seed treatment)	*Heterodera glycines*	*Microsclerotia seed inoculation*	Greenhouse trials	Decreased cyst nematode population (select isolates)	Greenhouse/pot	Silva et al. (2022)
*Trichoderma harzianum*	Potato	*M. incognita*	*Antagonism + enzymatic defense induction*	Open field	Reduced nematode population; ↑ antioxidant enzymes	Field	Elkelany et al. (2026)
*T. harzianum +* 1,3-D + organic matter	Tomato	*M. incognita*	*Combined chemical + biocontrol*	Field (2 consecutive crops)	Reduced nematode density + improved yield	Field	d'Errico et al. (2022)
*T. viride, T. harzianum* (sole/combined)	Potato	*M. incognita*	*Antagonism*	Field	78.7–83.7% nematode reduction (sole Trichoderma)	Field	Youssef et al. (2023)
*Trichoderma asperellum + Purpureocillium lilacinum*	Coffee (mature Arabica trees)	*M. hapla* (first report on coffee, Kenya)	*Soil drench application*	7 field trials, 2 years	Reduced nematode populations; altered soil nematode community	Field	Saikai et al. (2023)

SEM, Scanning Electron Microscopy; GC-MS, Gas Chromatography–Mass Spectrometry; CRD, Completely Randomized Design; J2, Second-stage juvenile (nematode); VOCs, Volatile Organic Compounds; BCA, Biological Control Agent; PGPR, Plant Growth-Promoting Rhizobacteria.

The reasons for this attrition between promising candidates and field-ready products are increasingly well documented. Field establishment is one major challenge. In Kenyan coffee systems both *P. lilacinum* and *T. asperellum* successfully colonized roots endophytically and persisted in the soil, but their establishment was only detected six months after application. This finding highlights how slowly and unpredictably endophytic colonization may develop under field conditions, compared with the rapid establishment often observed in short-term greenhouse experiments. Interactions with the resident microbiota can further modify outcomes. In tomato, *Piriformospora indica* contributed more strongly than either of two co-inoculated plant growth-promoting rhizobacteria to the suppression of *M. incognita*; however, its efficacy declined when all three microorganisms were combined, apparently because of antagonistic interactions between the fungal and bacterial partners (Varkey et al., 2018). Thus, increasing the number of beneficial microorganisms does not necessarily result in additive, and certainly not necessarily synergistic, effects.

Even when field efficacy is demonstrated, additional hurdles must be overcome before a fungal candidate can become a marketable product. Formulation must maintain fungal viability and stability during storage while remaining compatible with existing application equipment and agricultural practices. Moreover, although regulatory pathways for microbial biocontrol agents may be more streamlined than those for synthetic nematicides, registration still requires substantial efficacy, environmental, and safety data. Generating these datasets across multiple crops, environments, and seasons is resource-intensive and often beyond the capacity of academic research groups alone. Consequently, only a small proportion of fungal species reported to exhibit nematicidal activity have progressed beyond experimental evaluation to commercial products, with existing products remaining concentrated around a handful of well-characterized strains. Closing this laboratory-to-field gap will therefore require a shift from discovery-driven screening toward a more integrated development pipeline combining fungal ecology, formulation science, application technology, and multi-site field validation. In this context, the priority may be less to discover ever more fungal candidates than to understand why promising strains succeed or fail under field conditions and to engineer formulations and application strategies that make their performance more predictable, scalable, and economically viable (**Figure 5**).

**Figure 5:**
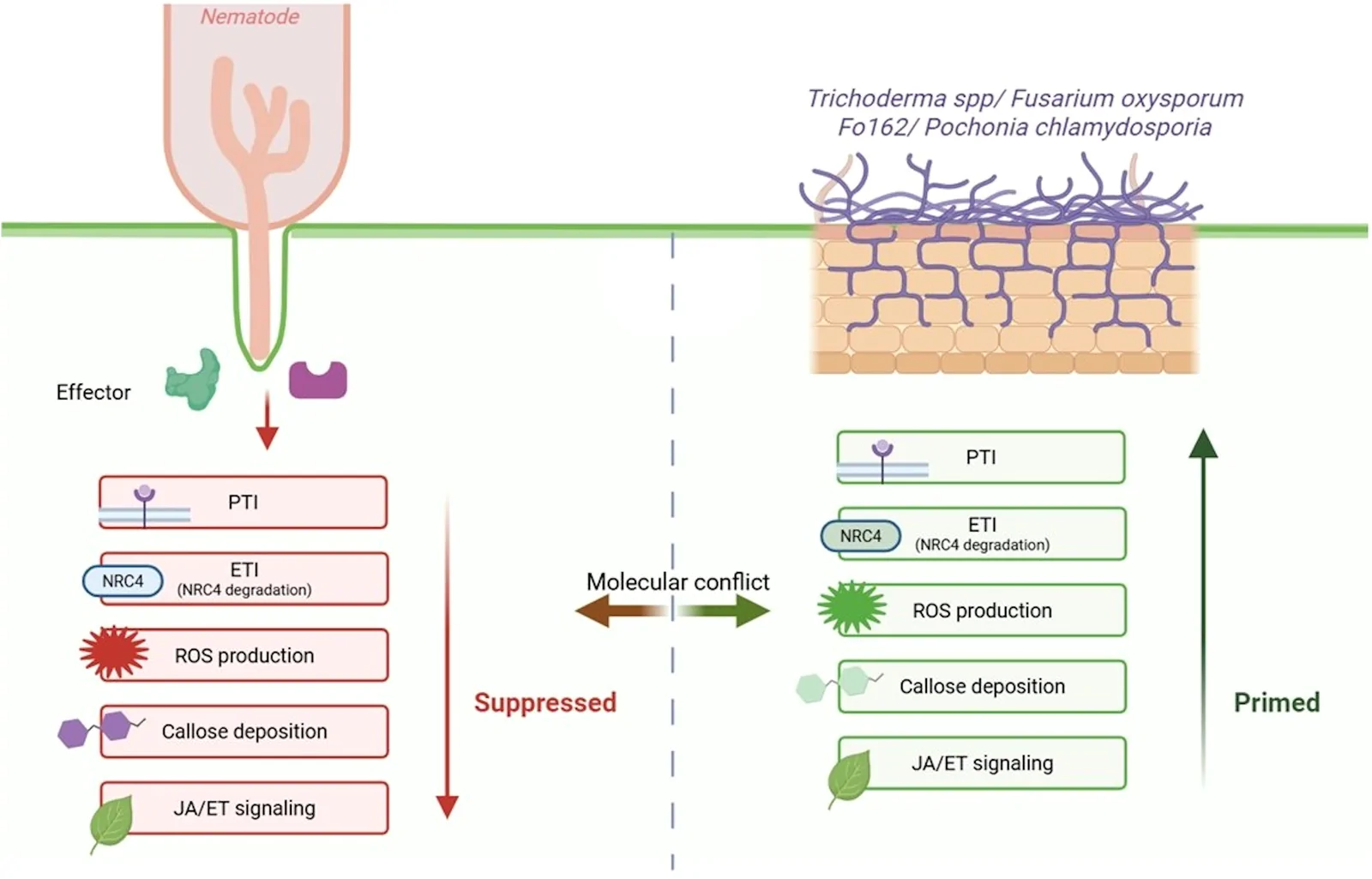
From laboratory to commercial product: the developmental pipeline of fungal bionematicides. Candidate endophytic fungi progress through five sequential stages—*in vitro* screening, greenhouse validation, multi-site field trials, formulation and registration, and commercial release—with substantial attrition occurring at each step due to declining efficacy under increasingly realistic conditions, strain- and soil-dependent variability, and growing formulation and regulatory requirements. The timelines illustrate the developmental trajectories of four representative species: *Pochonia chlamydosporia* and *Purpureocillium lilacinum* (strain PL11) have progressed through the pipeline to reach commercial development and/or regulatory approval (e.g., EFSA endorsement for PL11), whereas *Arthrobotrys thaumasia* and *Tolypocladium cylindrosporum* have so far advanced only to greenhouse validation, with no field-scale evaluation reported to date (Purba et al., 2022; Kassam et al., 2021). Reported field efficacy among candidates fungal bionematicides ranges from 30–80%, reflecting strong context dependence associated with fungal isolate identity, soil properties, and crop species. Bottom panel: Major bottlenecks limiting progression through the development pipeline include inconsistent field establishment, interactions with native soil microbiota, formulation stability, and the cost and compelxity of regulatory approval. Created in https://BioRender.com.

## Future perspectives

Despite substantial progress, several interconnected knowledge gaps continue to limit the translation of endophyte-mediated nematode suppression into reliable agricultural applications.

First, the molecular mechanisms underlying endophyte-induced defense priming against sedentary plant-parasitic nematodes remain insufficiently understood. Although hormonal reprogramming involving jasmonic acid and ethylene has been documented, the extent to which endophytes influence the perception of NAMPs and ETI, particularly during interactions with root-knot nematodes, remain largely unresolved.

Second, many promising nematicidal metabolites, including chaetoglobosin A, ergovaline, and guignardone D, have been characterized primarily under *in vitro* conditions. Their biological activity, stability, and mode of action in the rhizosphere and within plant tissues therefore require validation under biologically and environmentally realistic conditions. Bridging this gap will be essential to determine whether metabolites identified under controlled laboratory conditions can contribute meaningfully to nematode suppression in agricultural systems.

Third, the often substantial delay between fungal application and successful root colonization under field conditions highlight a critical need to identify the ecological and physiological determinants of reliable endophytic establishment. Understanding how soil properties, resident microbial communities, host genotype, environmental conditions, and fungal traits interact to determine colonization success will be central to improving the consistency of endophyte-based interventions.

Finally, translating promising greenhouse results into robust field performance will require a deliberate shift from discovery-driven screening toward integrated development pipelines. Such pipelines should combine mechanistic understanding of fungal ecology and plant–microbe interactions with advances in formulation science, application technology, quality control and multi-environment validation.

In conclusion, realizing the potential of endophytic fungi as reliable components of sustainable nematode management will depend not simply on discovering additional promising strains, but on developing a mechanistic understanding of why particular strains succeed or fail under field conditions. Equally important will be the development of formulations and application strategies that enhance the predictability, consistency, scalability, and economically viability of their performance across diverse agricultural environments.

## Figures and Tables

**Figure 6:**
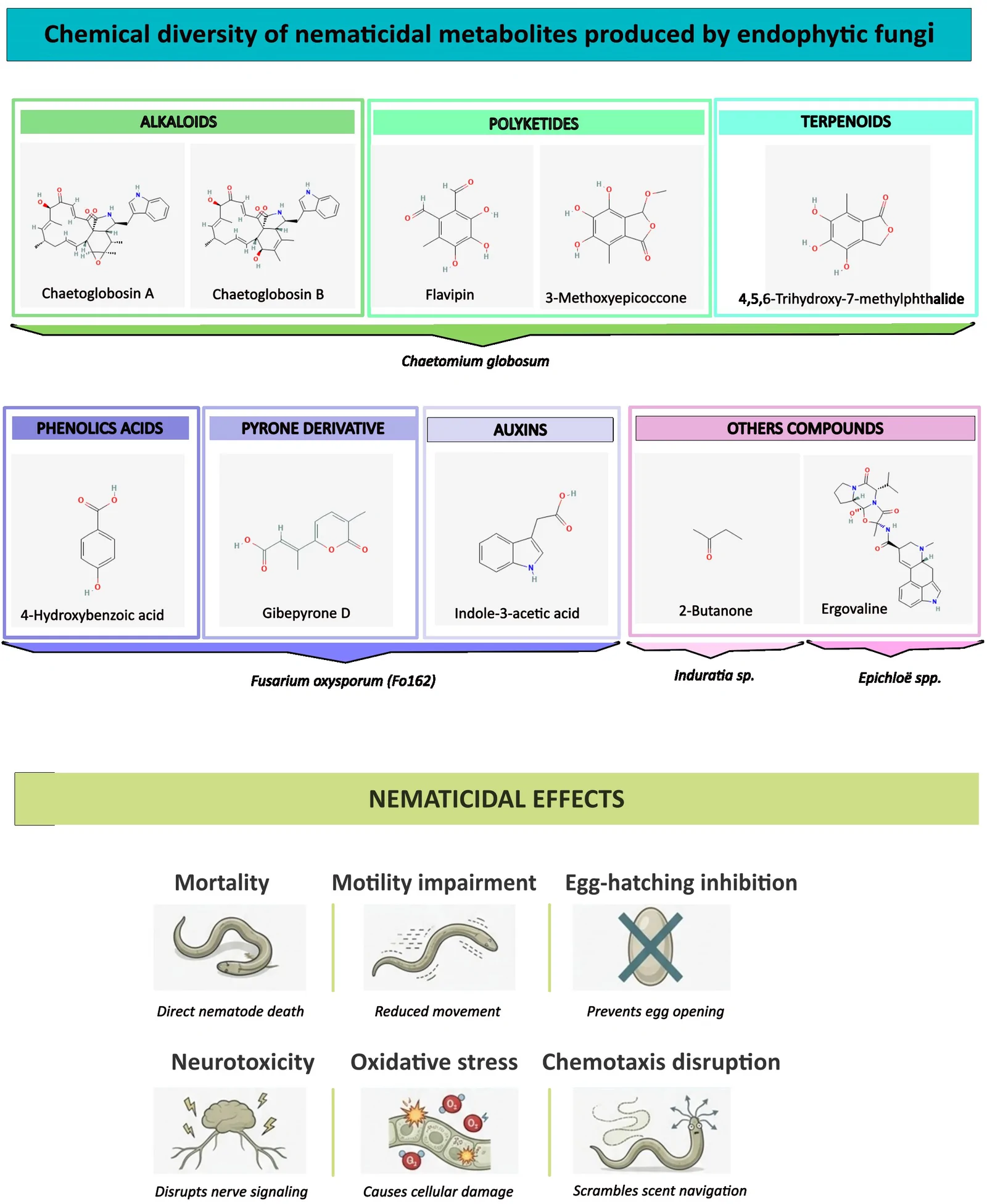
Chemical diversity of nematicidal metabolites produced by endophytic fungi and their principal modes of action. Upper panels show representative nematicidal compounds grouped by chemical class, alkaloids (chaetoglobosin A, PubChem CID 6438437; chaetoglobosin B, CID 12011905), polyketides (flavipin, CID 3083587; 3-methoxyepicoccone, CID 139586575), terpenoids (4,5,6-trihydroxy-7-methylphthalide, CID 10420195), produced by Chaetomium globosum; phenolic acids (4-hydroxybenzoic acid, CID 135) and pyrone derivatives (gibepyrone D, CID 20056005) produced by Fusarium oxysporum (strain Fo162); and other bioactive compounds, including the auxin indole-3-acetic acid (CID 802), 2-butanone (methyl ethyl ketone, CID 6569; Induratia sp.), and ergovaline (CID 104843; Epichloë spp.). Lower panel summarizes the principal nematicidal effects reported for these and related fungal metabolites: direct mortality, motility impairment, inhibition of egg hatching, neurotoxicity, induction of oxidative stress, and disruption of chemotaxis-mediated host location. Chemical structures were retrieved from the PubChem database (National Center for Biotechnology Information, U.S. National Library of Medicine; https://pubchem.ncbi.nlm.nih.gov). Together, these mechanisms illustrate the multiple modes of action through which endophytic fungal metabolites may contribute to nematode suppression. The figure was assembled in Inkscape (Inkscape Project); nematode illustrations were generated with Gemini (Google).
